# Prognostic significance of blood inflammatory biomarkers NLR, PLR, and LMR in cancer—A protocol for systematic review and meta-analysis

**DOI:** 10.1097/MD.0000000000014834

**Published:** 2019-06-14

**Authors:** Chellan Kumarasamy, Shanthi Sabarimurugan, Royam Madhav Madurantakam, Kartik Lakhotiya, Suja Samiappan, Siddhratha Baxi, Ramesh Nachimuthu, Kodiveri Muthukaliannan Gothandam, Rama Jayaraj

**Affiliations:** a University of Adelaide, North Terrace Campus, Australia; b School of Biosciences and Technology, Vellore Institute of Technology (VIT), Vellore; c Department of Biochemistry, Bharathiyar University, Coimbatore, Tamil Nadu, India; d Genesis Cancer Care Centre, Bunbury, Western Australia; e College of Health and Human Sciences, Charles Darwin University, Darwin, Australia.

**Keywords:** cancer, meta-analysis, MLR, NLR, PLR, protocol, systematic review

## Abstract

**Background:**

The neutrophil-lymphocyte-ratio, platelet-lymphocyte-ratio, and monocyte-lymphocyte-ratio have been explored as a simple, inexpensive, and effective method for cancer prognosis. However, there are no studies that have investigated the comparative utility of these markers, in multiple cancers.

**Methods:**

The preferred reporting items for systematic review and meta-analysis protocols (PRISMA-P) guidelines were used to design this meta-analysis protocol. The final study will also be conducted under the PRISMA guidelines for systematic reviews and meta-analyses. The core bibliographic database search will be carried out by 2 reviewers working individually, with each conducting an initial screening based on titles and abstracts. The shortlisted articles will be selected for review and quantitative analysis, based on predefined inclusion and exclusion criteria. Study characteristics, relevant clinicopathological characteristics, and statistical data required for meta-analysis (hazard ratios [HRs] and 95% confidence intervals [CIs]) will be extracted and compiled into a MS Excel datasheet. Meta-analysis will be performed, using a random-effects model, and the results (pooled HR and 95% CI) will be presented in the form of a forest plot. Publication bias will also be assessed by use of Egger bias indicator test and funnel plot symmetry. If statistical data from included studies is insufficient, a qualitative literature review will be pursued.

PROSPERO registration: PROSPERO CRD42019121008.

## 1 Introduction

Tumor microenvironment plays a critical role in tumorigenesis and cancer cell growth.^[1]^ One of the major hallmarks of cancer, which plays a role in tumor malignancy, angiogenesis, genome instability, as well as systemic alterations, is inflammation.^[2]^ Inflammation and inflammatory markers, when associated with cancer have been shown to lead to a worse prognosis.^[3]^ Therefore, assessing the magnitude of inflammation in cancers may allow us to use it as a clinical prognostic marker. This magnitude of inflammation can be captured by the measurement of the neutrophil-lymphocyte ratio (NLR), platelet-lymphocyte ratio (PLR), and monocyte-lymphocyte ratio (MLR). As systemic alteration of inflammation leads to alteration in the peripheral blood leukocytes, NLR can act as a proxy measurement of the degree of inflammation in cancers.^[4,5]^ Similarly, platelets release pro-inflammatory mediators, such as cytokines and chemokines, which exacerbate the inflammatory microenvironment in tumors, making PLR another viable measure of inflammation.^[6,7]^ Monocytes have also been observed to have a key role in inflammation, also having been implicated in many inflammatory diseases, including atherosclerosis.^[8]^

The measurement of NLR, PLR, and MLR is simple, rapid, and inexpensive, while simultaneously being associated with little to no patient discomfort, (as only peripheral blood samples are required for testing).^[9]^ Current methods for cancer prognosis in patients involves the use of molecular markers (such as BRCA1 in breast cancer and epidermal growth factor receptor in non-small-cell lung carcinoma),^[10,11]^ which require complex and expensive assays for measurement and quantification (immunohistochemistry, q-RT PCR)^[12,13]^, while also generating a greater degree of patient discomfort (biopsy). Therefore, a great deal of research interest has been directed towards the use of PLR, NLR, and MLR as biomarkers for cancer prognosis.

Multiple clinical studies have explored the use of PLR, NLR, and MLR, not only in cancers, but also as preoperative prognostic biomarkers.^[14,15]^ This abundance of literature has also led to the publication of multiple systematic review and meta-analysis studies regarding this topic.^[16,17]^ However, no systematic review or meta-analysis study published till-date, has investigated;

(1) The prognostic efficacy of PLR, NLR, and MLR in comparison to each other, with regards to cancer,
(2) The difference in prognostic efficacy of PLR, NLR, and MLR, across various types of cancers.

Previous systematic-review and meta-analysis studies have only focused on a single cancer type, with no study conducting a comparative analysis of PLR, NLR, and MLR in cancer. Mellor et al recently published a systematic review and meta-analysis study, including all cancer types, but only focusing on NLR alone as the prognostic marker of choice.^[18]^ Similarly, Zhu et al focused on multiple inflammatory markers, with emphasis on PLR and NLR, but limited their study to ovarian cancer,^[19]^ while Zhang et al limited their analysis to colorectal cancer.^[16]^

This proposed systematic review and meta-analysis study intends to amend this existing knowledge gap, and provide a better understanding of the utility of PLR, NLR, and MLR as prognostic markers in cancer, while simultaneously attempting to highlight the best marker (amongst PLR, NLR, and MLR), with respect to each type of cancer.

## 2 Methods

### 2.1 Search strategy

The proposed systematic review and meta-analysis study follows the standard guidelines as established in preferred reporting items for systematic review and meta-analyses protocol (PRISMA-P) guidelines.^[20,21]^ The search strategy is designed to be expansive and exhaustive, with the bibliographic databases, EMBASE, MEDLINE, Science Direct, Scopus, and Web of Science, acting as sources of published literature. The literature search will be limited to the past decade (1998–2018) to maintain the relevance of the study to current scope of research regarding cancer prognosis. The search strategy will also allow for extraction of relevant literature from reference lists of shortlisted articles to further increase the robustness of the search. The search will be carried out based on “search strings” designed from subject-relevant “keywords” (depicted in Table 1). Initial search and screening will be performed by 2 reviewers, with the initial screening based on relevance of each published study to the proposed systematic review and meta-analysis, as can be observed from the information available in the titles and abstracts. The screening of titles and abstracts will be left to the expertise of 2 reviewers. A final unbiased verification will be conducted by a 3rd reviewer, after which the full texts of studies screened-in, will be subject to rigorous selection criteria.

**Table 1:**
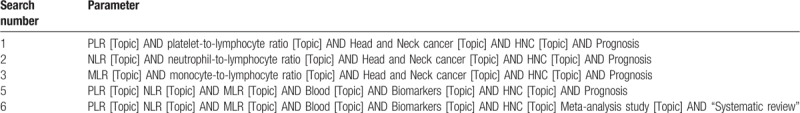
The initial search strategy PubMed.

### 2.2 Study selection

After initial screening, the full-texts of articles will be subject to selection based on the following inclusion and exclusion criteria. The inclusion and exclusion criteria are based on previous similar systematic review and meta-analysis studies, and have been adopted to the specifications of this current proposed study.

### 2.3 Inclusion criteria

(1) The studies must discuss the survival outcome of cancer patients based on PLR, NLR, and MLR levels.
(2) The survival outcome must be presented in the form of hazard ratios (HR) and 95% confidence intervals (CI).
(3) The survival outcome must be presented in the form of Kaplan–Meier (KM) curves, along with patient cohort information, for each treatment arm represented in the KM curves. (This requirement is only subject to condition, if the HR and 95% CI values have not been presented in manuscript, as the above information is required to extract approximated HR values).

### 2.4 Exclusion criteria

(1) Conference abstracts, reviews, letters to the editor, and other nonclinical literature will not be considered for either systematic-review or meta-analyses.
(2) Included studies must be clinical studies or involve patient samples. (in-vitro, in-silico, and animal studies will be excluded).
(3) Unpublished, or non-peer-reviewed literature will be excluded.
(4) Studies that do not focus on survival outcomes and prognosis aspects of PLR, NLR, and MLR in cancer patients.
(5) If the sample size of each individual study if of low power, (sample size <10) they will be excluded.
(6) There will be no limitations on the types of patients involved or clinicopathological parameters, as part of each included study. No limitations based on gender, age, ethnicity, location, follow-up period, duration of treatment or method of treatment will be placed, on the studies being included.

### 2.5 Data extraction and recording

After selection of studies, based on the predefined inclusion and exclusion criteria, the full-texts will be subject to a data extraction process. Data extraction will follow a top-down approach, with the selected full-texts being combed for relevant patient and study data, individually, by 3 reviewers (to generate redundancy, while reducing individual error). A standardized, data extraction form will be prepared using Microsoft Excel, and will be utilized by the reviewers to extract the data. After individual data extraction has been performed by all 3 reviewers, the collected dataset of study information will be collated into a single database (along with elimination of duplicated information/studies), which will be used to conduct further analyses.

### 2.6 Data items

The data items which will be extracted from the full-texts of studies, include;

(1) Author names
(2) Year of publication
(3) Marker studied (PLR, NLR, or MLR)
(4) Size of patient cohort
(5) Diagnostic methods.
(6) Follow-up period
(7) Gender split of cohort
(8) TNM staging split of cohort
(9) Survival endpoint of each study (overall survival [OS], disease-free survival [DFS], disease-specific survival [DSS])
(10) General features of each study (will be presented as short qualitative opinions/observations of reviewers, towards each study being included)

After data extraction, all selected articles will be input into EndNote, to form a bibliographic database, to help in future data processing.

### 2.7 Quality assessment

The quality assessment of the studies was based on the standard, Newcastle–Ottawa scale for quality assessment of nonrandomized studies in meta-analysis.^[22]^ This scale presents a “star system,” which assigns each of the 3 broad parameters of the study, 1 to 4 stars, in increasing order of quality. The 3 broad perspectives being assessed include, the selection of study groups in each individual study, the comparability of groups, and the ascertainment of exposure/outcome of interest for case–control/cohort studies.^[23–27]^ This tool offers a simple and succinct, yet comprehensive analysis of the quality of each included study, and allows us to visualize it with clarity.

### 2.8 Meta-analysis

The meta-analysis itself, will be conducted using the comprehensive meta-analysis software (version 3.3.070; Biostat, Englewood, NJ).^[28]^ The software will be used to generate forest plots representing the meta-analysis results. The HR effect size metric, will be pooled across all included studies to determine the prognostic utility of PLR, NLR, and MLR in cancer. The HR effect size metric, indicates the survival probability of the patient cohort in each individual study, and the pooled HR will determine the likelihood of survival across all studies. The HRs of OS, DFS, and DSS, from each study, will be pooled separately, as each indicate a different type of survival endpoint. Similarly, meta-analysis on PLR, NLR, and MLR will be conducted individually, to assess the prognostic utility of each as a marker in cancer. The statistical significance (*P*-value) of PLR, NLR, and MLR, will also be assessed, based on the meta-analysis of HR values. Statistical significance allows us to verify whether the pooled results accept or reject the null hypothesis (in the case of this study, it translates to if PLR, NLR, and MLR high/low values, lead to better/worse survival in all cancer patients), while the effect size metric, informs us regarding the magnitude of the effect (in the case of this study, it indicates the % by which the pooled values fall to either side of the null hypothesis), which allows us to measure the prognostic utility of PLR, NLR, and MLR in cancer. All analyses will be carried out using the random-effects model, owing to the between study heterogeneity that is inherent when comparing individual studies and publications.^[29]^

### 2.9 Assessment of heterogeneity

The heterogeneity between studies will be assessed based on 3 parameters.^[30–34]^ The Higgins *I*^2^ statistic will be used as the primary method to determine heterogeneity, as it has a high power of detection of heterogeneity.^[35]^ However, *I*^2^ is not an infallible metric of heterogeneity, as it can provide biased results in small meta-analysis.^[36]^ Therefore for redundancy, we will use the Cochran’ *Q* and Tau^2^ parameters to assess heterogeneity alongside the *I*^2^ statistic.^[37,38]^

### 2.10 Subgroup analysis

The major meta-analysis subgroups for this study are studies which assessed PLR, NLR, and MLR. Analysis of other subgroups is contingent upon the availability of high-quality statistical data from included studies. If sufficient data is available, the 3 different survival endpoints, OS, DFS, and DSS will be analyzed separately, (otherwise, the meta-analysis will only assess OS). For additional subgroup analysis, cancer type, location, ethnicity, and follow-up period will be considered.

## 3 Publication bias

Assessment of publication bias is integral for validating the results of any meta-analytic study.^[21,39–42]^ Publication bias is caused as a natural side effect of the publication process, wherein small studies and negative results are rarely published as part of peer-reviewed literature. Therefore, it is important to perform analysis of publication bias, so as to understand the results of the meta-analysis, in context to the bias existing within the study.^[26,32]^

Egger graphical bias indicator test will be used to construct a funnel plot.^[43]^ The symmetry of the funnel plot along the regression line will offer a simple method to assess the publication bias. The symmetry of the funnel plot is inversely related to the degree of publication bias in the study.

Orwin Fail-Safe *N* test will allow us to adjust the funnel plot for small missing studies.^[44]^ This method allows for imputation of small studies in the plot, to more accurately assess the bias present.

Begg and Mazumdar Rank Correlation test will be used to check correlation between ranks of effect sizes and their respective variances.^[45]^ A positive result on this test indicates that the publication bias assessment is accurate.

## 4 Discussion

PLR, NLR, and MLR have great potential for clinical use as prognostic markers in cancer. However, previous studies have been limited in scope to single cancer types. Furthermore, no study has performed any comparative assessment of the aforementioned markers. This limitation is not only observed in the individual clinical studies, but also in recent systematic reviews and meta-analysis.^[46,47]^ Therefore, there is a requirement for a study that explores the overall picture of PLR, NLR, and MLR as prognostic markers, across all cancer types. The systematic review and meta-analysis format of study, in particular, allows us to pursue this goal, as the study is based on the collation of all published research in this field. This protocol will allow us to conduct such a study, in a precise manner while ensuring that the present research in this field is accurately represented in its results.

The tentative results of the proposed study should be able to inform us regarding the extent clinical significance of each prognostic marker in particular types of cancers. It will also indicate the areas, where further research is necessary, before clinical application of PLR, NLR, and MLR as cancer markers can be considered.

Therefore, this study will have clinical relevance by suggesting the best markers across PLR, NLR, and MLR in each particular type of cancer, or even in the case of negative results, support and guide future research by highlighting areas where research is required for establishing PLR, NLR, and MLR as cancer markers.

Cancer research is an ever-evolving field, and as more literature continues to be published, the protocol will also continue to be relevant in the future, as the study may require to be update to incorporate ongoing advancements in this field. Therefore, this protocol will allow for standardization of the process of conducting of an updated study, while also tangentially benefiting any future researchers conducting a systematic review or meta-analysis study regarding cancer biomarkers.

### 4.1 Reporting of the review

**Figure 1:**
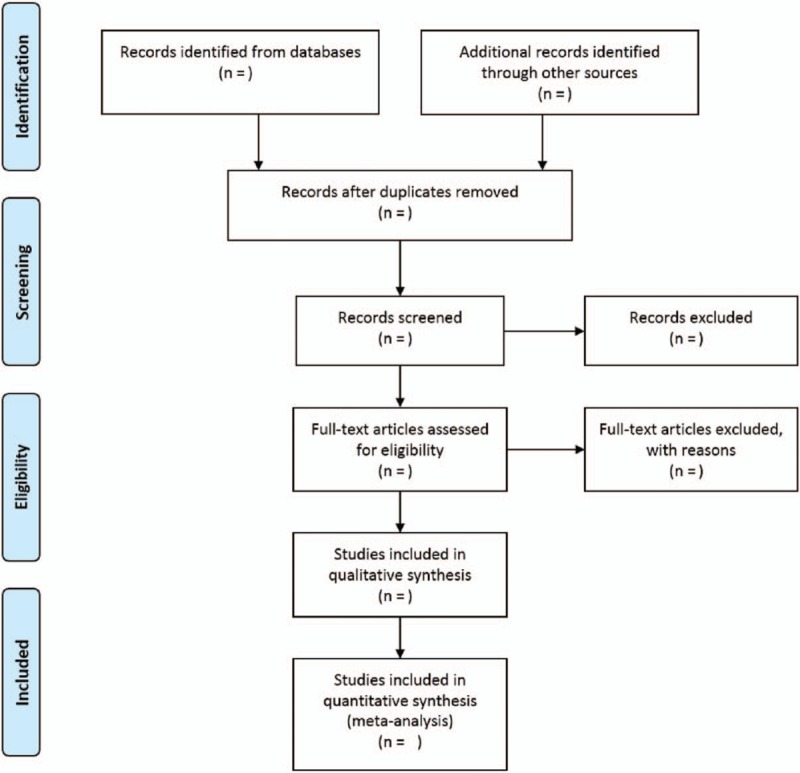
Diagrammatic representation of the flow chart of the articles to be selected.

The findings will be published as per PRISMA guidelines.^[48]^ A flow chart will be employed to outline the selection process (Fig. 1). Text description will be used to review the qualitative data of the included studies. Outputs of meta-analyses will be depicted in a forest plot. Publication bias will be represented in the inverted funnel plot. The search strategy and quality appraisal tool will be provided in the supplement.

### 4.2 Ethics and dissemination

This protocol is prepared according to PRISMA-P guidelines.^[20]^ This study will be conducted using publicly available data without involving human participants. Therefore, it does not require formal human research ethics committee review. We plan to publish our findings in peer-reviewed journals and relevant conference proceedings. In addition, we believe the results of the systematic review will have implications for policy and practice. We will prepare policy-maker summary using a validated format, disseminate through social media, and email discussion groups.

## Acknowledgments

We would like to acknowledge the Meta-analysis concepts and applications workshop manual by Michael Borenstein for his guidelines on reporting Meta-analysis, subgroup analysis and publication bias, (www.meta-analysis-workshops.com).

## Author contributions

RJ, CK, MRM, and SS contributed to the conceptualization, study design, search strategy, protocol development, and review by revising different versions. RJ, SB, SS, RN, KMG were involved in the supervision, ensured the absence of errors, and arbitrated in case of disagreement. CK, SS, MRM, KL engaged in the manuscript writing and analysis. All authors have read and approved the final version of the manuscript.

**Conceptualization:** Chellan Kumarasamy, Madhav Madurantakam Royam, Rama Jayaraj.

**Data curation:** Chellan Kumarasamy, Shanthi Sabarimurugan, Rama Jayaraj.

**Formal analysis:** Chellan Kumarasamy, Shanthi Sabarimurugan, Madhav Madurantakam Royam, Rama Jayaraj.

**Investigation:** Chellan Kumarasamy, Rama Jayaraj.

**Methodology:** Chellan Kumarasamy, Kartik Lakhotiya, Suja Samiappan, Rama Jayaraj.

**Project administration:** Shanthi Sabarimurugan, Gothandam K M, Rama Jayaraj.

**Resources:** Madhav Madurantakam Royam, Suja Samiappan, Siddhartha Baxi, Rama Jayaraj.

**Software:** Rama Jayaraj.

**Supervision:** Siddhartha Baxi, Ramesh Nachimuthu, Gothandam K M, Rama Jayaraj.

**Validation:** Kartik Lakhotiya, Ramesh Nachimuthu.

**Writing – original draft:** Chellan Kumarasamy, Shanthi Sabarimurugan, Madhav Madurantakam Royam, Kartik Lakhotiya, Suja Samiappan, Ramesh Nachimuthu, Gothandam K M, Rama Jayaraj.

**Writing – review and editing:** Chellan Kumarasamy, Shanthi Sabarimurugan, Madhav Madurantakam Royam, Kartik Lakhotiya, Suja Samiappan, Siddhartha Baxi, Ramesh Nachimuthu, Gothandam K M, Rama Jayaraj.

Rama Jayaraj orcid: 0000-0002-2179-0510.
